# Maturation of Brain Regions Related to the Default Mode Network during Adolescence Facilitates Narrative Comprehension

**DOI:** 10.4172/2375-4494.1000328

**Published:** 2017-01-12

**Authors:** Tzipi Horowitz-Kraus, Rola Farah, Ardag Hajinazarian, Kenneth Eaton, Akila Rajagopal, Vincent J Schmithorst, Mekibib Altaye, Jennifer J Vannest, Scott K Holland

**Affiliations:** 1 Educational Neuroimaging Center, Faculty of Education in Science and Technology, Technion, Israel; 2 Reading and Literacy Discovery Center, Cincinnati Children’s Hospital Medical Center, Cincinnati, Ohio, USA; 3 Pediatric Neuroimaging Research Consortium, Cincinnati Children’s Hospital Medical Center, University of Cincinnati, Cincinnati, Ohio, USA; 4 Communication Sciences Research Center, Cincinnati Children’s Hospital Medical Center, Cincinnati, Ohio, USA; 5 Department of Radiology, Children’s Hospital of Pittsburgh of UPMC, Pittsburgh, Pennsylvania, USA

**Keywords:** Children, Default mode network, Functional magnetic resonance imaging, Narrative comprehension, Language

## Abstract

**Objectives:**

Although the Default Mode Network (DMN) has been examined extensively in adults, developmental characteristics of this network during childhood are not fully understood.

**Methods:**

In this longitudinal study, we characterized the developmental changes in the DMN in fifteen children who were each scanned three times during a narrative comprehension task using magnetic resonance imaging.

**Results:**

Despite similar brain-activation patterns along developmental ages 5 to 18 years when listening to stories, increased, widely distributed deactivation of the DMN was observed in children between the ages of 11 and 18 years. Our findings suggest that changes occurring with increased age, primarily brain maturation and cognitive development drive deactivation of the DMN, which in turn might facilitate attendance to the task.

**Conclusions:**

The interpretation of our results is as a possible reference for the typical course of deactivation of the DMN and to explain the impaired patterns in this neural network associated with different language-related pathologies.

## Introduction

Narrative comprehension is one of the first linguistic abilities to be acquired during development, and it is defined as the ability to understand and comprehend information that is presented orally, particularly when listening to stories [1]. Language, as it is understood at the story level, likely depends on complex interactions between the language neural system and other cognitive domains [2–4]. Szaflarski and colleagues [4] characterized the developmental trajectory of narrative comprehension in school-age children. In addition to increased activation in brain regions related to auditory and language processing [i.e., the superior temporal gyrus (STG)], the authors reported age-related decreases in cortical activation in regions identified within the Default Mode Network (DMN) [5,6].

The DMN is considered a distinct functional brain network supporting cognitive control and reflecting neural activity at rest or in the absence of an extraneous stimulus [6], or when individuals are engaged in self-referential thoughts [7]. In adults, the DMN physiologically includes the medial prefrontal cortex (MPFC), posterior cingulate cortex (PCC), inferior parietal lobule, precuneus, anterior cingulate cortex (ACC), inferolateral temporal cortex, lateral parietal cortex, and hippocampal formation [7–10]. In school-age children, a similar network has been identified with some reports of decreased activation in prefrontal regions and the inclusion of additional regions in the postcentral gyrus, insula, and inferior occipital regions compared to adults [11].

Although the DMN is typically known as an active network during rest, a large body of research has indicated the importance of its deactivation during the performance of cognitively demanding tasks; greater cognitive demand was associated with greater magnitude of DMN deactivation in an adult population [5,10,12–14]. Furthermore, increased activity in the DMN has been reported during high-level social cognitive tasks [15].

The exact functions of the DMN are still largely unknown, but brain regions included in this network are known to be involved in the integration of self-monitoring and autobiographical, memory, and social cognitive functions [16]. Deactivation of the DMN is associated with attention abilities based on findings that deficient deactivation can result in attention lapses and consequently a failure to maintain a goal-directed behavior [17,18]. Whether we are pondering our old memories, brainstorming for new ideas, or paying attention to a given stimulus, the DMN plays a key role in those processes. The current study was designed to examine the longitudinal changes in deactivation of regions related to the DMN starting from childhood to adolescence during one of the most basic, innate human abilities: a narrative comprehension task.

Most studies demonstrating the existence of the DMN have been conducted in adult populations. More recently it appears that the same general pattern for the DMN in adults is also found in children ranging in age from 7 to 9 years [19], as well as in 7–12 year-old children [11]. Despite the existence of similar nodes within the DMN in children, Supekar and colleagues found evidence for reduced functional connectivity between some of the nodes in children when compared to young adults. In children, several DMN components showed weak functional connectivity and exhibited significant changes developmentally [19]. Weakly connected regions in children included the PCC and the MPFC during a resting-state condition [20]. Although some researchers attributed this weaker functional connectivity to differences in motion along age (i.e., greater motion artifacts in younger vs older individuals), we did not find any of age on motion in our sample [20]. Cabeza and colleagues suggested that the brain of an older individual may utilize different brain regions than those of a younger one during a cognitive task, due to frontal maturation and functional reorganization [21]. An examination of the developmental changes in the deactivation of regions related to the DMN during fluency and executive functions tests reveals that as an individual becomes older, the functional connectivity of the DMN becomes stronger and more connected to the cingulum bundle [22,23]. Similar immature structural connectivity was revealed using Diffusion Tensor Imaging or DTI tractography in tracts that connect the PCC and the MPFC in children compared to adults [19,24]. The authors postulated that their results reflect possible myelination processes and continued structural organization of axonal tracts, typically seen early in development to late adulthood [25]. In contrast, weaker functional connectivity between the PCC and the MPFC in younger children is thought to be a result of motion artifacts ([20] among others). Whether or not these changes along development affect the deactivation of the DMN and how this relates to one of the basics linguistic abilities, such as the ability to process orally presented stories, is still unknown.

The aim of the current study was to investigate the course of DMN deactivation as the brain matures by examining patterns of deactivation during a task that measures a basic linguistic ability - a narrative comprehension task [26]. Fifteen children ages 5–18 years participated in a narrative comprehension fMRI task three times over a 10year span (the same participants were scanned three times at approximate ages 5–7, 11, and 18 years). We hypothesized that due to the reported cognitive and neurophysiological changes accompanying brain maturation in this age-range and the involvement of the DMN in these cognitive abilities, regions related to the DMN would show increased deactivation throughout development accompanied by improved behavioral performance.

## Materials and Methods

### Participants

Participants in the current study were fifteen children (8 female, 7 male) who were previously recruited for a large-scale cross-sectional study of language development [26] and continued in a longitudinal component of the study from 2000 until 2012 [4]. All participants were followed and tested at three time points during their development (at approximate ages 5–7, 11, and 18 years). The data was assigned to three age groups: Test1-youngest (n=15, mean age=6.53 ± 0.99 years), Test2-middle (n=15, mean age=11.53 ± 1.59 years) and Test3-oldest (n=15, mean age=17.53 ± 1.30 years) groups. Our longitudinal sample population (n=15 subjects) were a subset of the larger study who successfully completed scanning at these three age groups. All participants were right handed, native monolingual English speakers, and with no history of neurological or psychiatric disorder or learning disabilities. All participants older than 11 years of age gave informed written assent and all parents provided informed written consent for all children enrolled prior to inclusion in the study, and all were compensated for their participation. The Cincinnati Children’s Hospital Medical Center (CCHMC) Institutional Review Board approved the study.

## Procedure

### Behavioral measures

To verify normal verbal and non-verbal IQ, the Wechsler Intelligence Scale for Children (WISC-3:Kaufman [27]) was administered to all children at entry into the study in 2000–2002.

The narrative comprehension task entailed a 30-s, short, complete story read by an adult, female speaker in an on-off block design (a transcript of one of the stories is found in Schmithorst, et al [28]), audio tracks of the stories can be downloaded from: https://irc.cchmc.org/software/pedaudio.php). The stories included 9–11 sentences of varying syntactic construction (e.g., conjoined sentences, center embedding) including complex syntactic structure to increase the relative processing load for this aspect of language. All stories were identical for all ages and were presented fully, without interruption. The control condition consisted of 1 second duration pure tones presented in an unequal interval of 1–3 s during the 30-s control period and was designed to control for sub-lexical auditory processing. Different tone frequency was presented (150, 200, 250, 500, 700, 900, or 1000 Hz) and changed randomly. Participants listened to 30-s blocks of story presentations interleaved with 30-s blocks of tones and were instructed to listen to the stories carefully in order to answer questions about them outside the scanner after the procedure.

After completing the scan, participants in all groups were asked to answer two multiple-choice questions about each story, for a total of ten questions covering the five stories heard. A language specialist designed the stories used in this study to ensure meaningful comprehension of those stories by all participants in the study. None of the stories contained elements of the theory of mind [29,30].

The purpose of the post-hoc testing was to verify that participants listened to the stories during the fMRI scan and also to verify comprehension (see [28] for examples). Measuring comprehension after completion of the scan, without taking a break, allowed the stories to be heard without interruption and more closely resemble the natural course of listening comprehension. Similar brain activation patterns [1] and similar functional network connectivity [31] have been indicated when comparing on-line and off-line versions of this task, which allowed the use of the off-line narrative comprehension task without concern of it affecting the results.

To verify the differences in narrative comprehension between the groups across development, RM-ANOVA was conducted.

### MRI acquisition and data preparation

MRI scans were obtained using a Bruker 30/60 Medspec imaging system (Bruker Medizintechnik, Karlsruhe, Germany). For stimuli and movie presentation, during the preparation (e.g., shimming) and for acquisition of the whole-brain anatomical scans, an MRI compatible audio/visual system (Avotec, SS3150/SS7100) was used. A gradient echo, EPI sequence was used for T2*-weighted BOLD fMRI scans with the following parameters: TR/TE=3000/38 ms; BW=125 kHz; FOV= 25.6 × 25.6 cm; matrix=64×64; slice thickness=5 mm. Twenty-four axial slices covered the entire cerebrum. 110 scan volumes were acquired during each fMRI experiment, consisting of five on/off cycles for 30 s per condition, for a total acquisition time of 5 min and 30 s. The first ten images were discarded to allow the spins to reach relaxation equilibrium. Participants were acclimated and desensitized to the scanner to condition them for comfort [32]. Head motions were minimized by using elastic straps, attached to either side of the head-coil apparatus to hold the head in place. T1-weighted, anatomical MRI scans were obtained for co-registration using a modified driven equilibrium Fourier transform method or MDEFT [33]. Data was analyzed using in-house processing software written in Interactive Data Language. Data was corrected for Nyquist ghosts and geometric distortion using the multi-echo reference method [34] and motion-corrected using pyramid co-registration [35]. A three-dimensional affine transformation was performed to align the volumes, yielding six motion parameters that were included as regressors in the first-level General Linear Model analysis. Furthermore, time points with excessive motion were rejected from the post-processing pipeline. We used a mutual information cost function for rejecting motion-corrupted frames of fMRI data [36]. All data met the criterion of median voxel displacement in the center of the brain < 2 mm (i.e., < 1/2 pixel). The fMRI data were transformed into stereotaxic space [37] using a linear affine transformation [38]. To rule out the effect of different age groups on motion level, a 3 × 6 RM-ANOVA (age group x motion axis) was performed to verify no age group x motion axis interaction. To verify the absence of motion effect within each axis, six RM-ANOVAs were performed corresponding with the six motion parameters (X, Y, Z; X rotated, Y rotated, Z rotated). After motion correction, normalization, and spatial smoothing, fMRI data were co-registered to a standard Talairach template. The use of the Talairach standard for children ages 5 years and older has been shown to produce minimal errors in co-registration for group analysis [39,40].

### Generation of group activation maps

To examine significant group activation and deactivation in the narrative comprehension>tone listening contrast, a general linear model and random-effects analyses were used. Images of the t-maps generated by this contrast were thresholded to P<0.001, Family Wise Error Rate or FWER corrected via Monte Carlo simulation [41].

In the group activation maps, the centroid of each region that survived significance the described statistical threshold was identified by examining all slices that contained suprathreshold voxels and manually selecting the axial slice (z coordinate) that was at the center of each cluster. Using the in-house program, we then outlined the ROI containing the cluster of activation/deactivation in the Test3-oldest group map and created a mask that was applied to the other two groups. The ROI tool computed the center of mass of the suprathreshold voxels within the ROI and returned the coordinates of this centroid as x, y, z values in the Talairach space.

### Imaging data analysis

To quantify the developmental change in deactivation of the DMN, we counted the number of suprathreshold voxels in each ROI in the mask (generated based on the deactivation pattern of the Test3-oldest group). We then included the number of voxels per ROI for each group in several separate RM-ANOVA. To determine the relationship between narrative comprehension ability and the deactivation in regions related to the DMN, the number of deactivated voxels was then correlated with narrative comprehension scores among the three age groups (N=45) while keeping the threshold constant [i.e., P<0.001 (FWER corrected)], after [42,43].

## Results

### Neuropsychological testing

Neuropsychological testing in the 5–7 years age group (i.e., Test1-youngest group) yielded an average IQ of 119.5 ± 13.4 (norm standard score is 100 ± 15).

### Narrative comprehension

The averaged correct responses for the verbatim narrative comprehension post-test outside the scanner was 77% (±14) for the Test1-youngest group, 82% (±10) for the 11 years age group (Test2-middle group), and 91% (±10) for the 18 years age group (Test3-oldest group). Repeated Measures Analysis of Variance (RM-ANOVA) revealed a significant difference in performance scores along development {F(2, 42)=4.8, P<0.05}. Post-hoc analysis suggested that this difference could be attributed to a significant difference between the narrative comprehension scores at 11 years old versus 18 years old (P<0.05), as well as at 5–7 years old versus 18 years old (P<0.01).

### The effect of age on motion

A 3 × 6 RM-ANOVA (age group x motion axis) revealed no significant interaction between age x motion interaction {F(10,4)=5.001, P>0.05}. The RM-ANOVA analyses revealed no significant effect for age on motion {X: F(2,14)=0.464, P=0.508, Y: F(2,12)=0.676, P=0.527, Z: F(2,12)=0.423, P=0.665, X rotated: F(2,12)=0.319, P=0.773, Y rotated: F(2,12)=1.564, P=0.249, Z rotated: F(2,12)=2.297, P=0.143}.

### Random effect analysis-fMRI results

**Figure 1:**
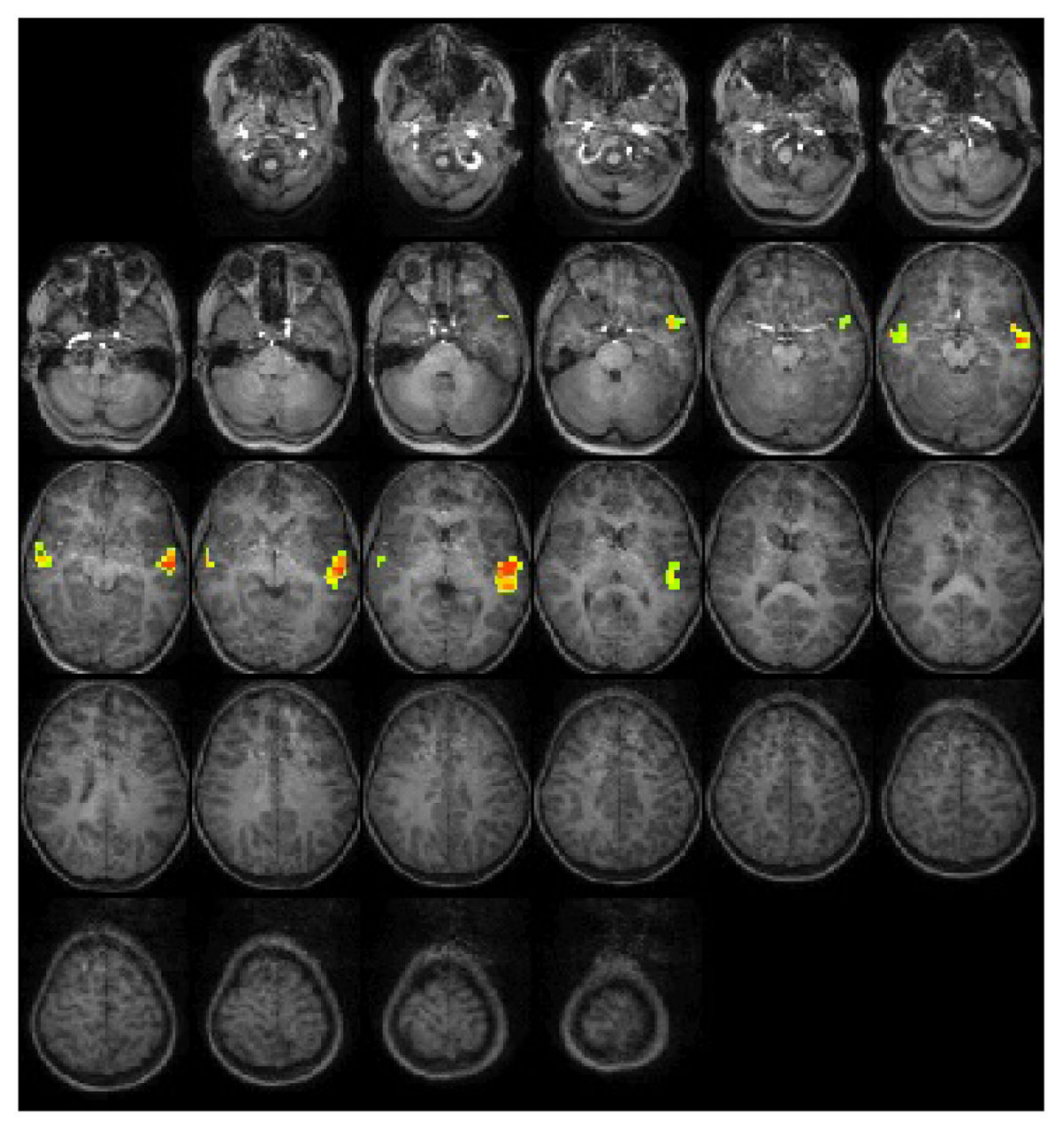
Group composite fMRI activation maps for narrative comprehension in the Test1-youngest group [5–7] year-old children.

For the Test1-youngest group (5–7 years): The statistical parametric map for narrative comprehension>tone listening (Figure 1) was consistent with previous studies using this task, demonstrating an increased activation in the superior frontal gyrus [26]. While significant positive activation was found in the left and right STG (BA 22), no significant deactivation was revealed. Talairach coordinates of cluster centroids are listed in Table 1.

**Figure 2:**
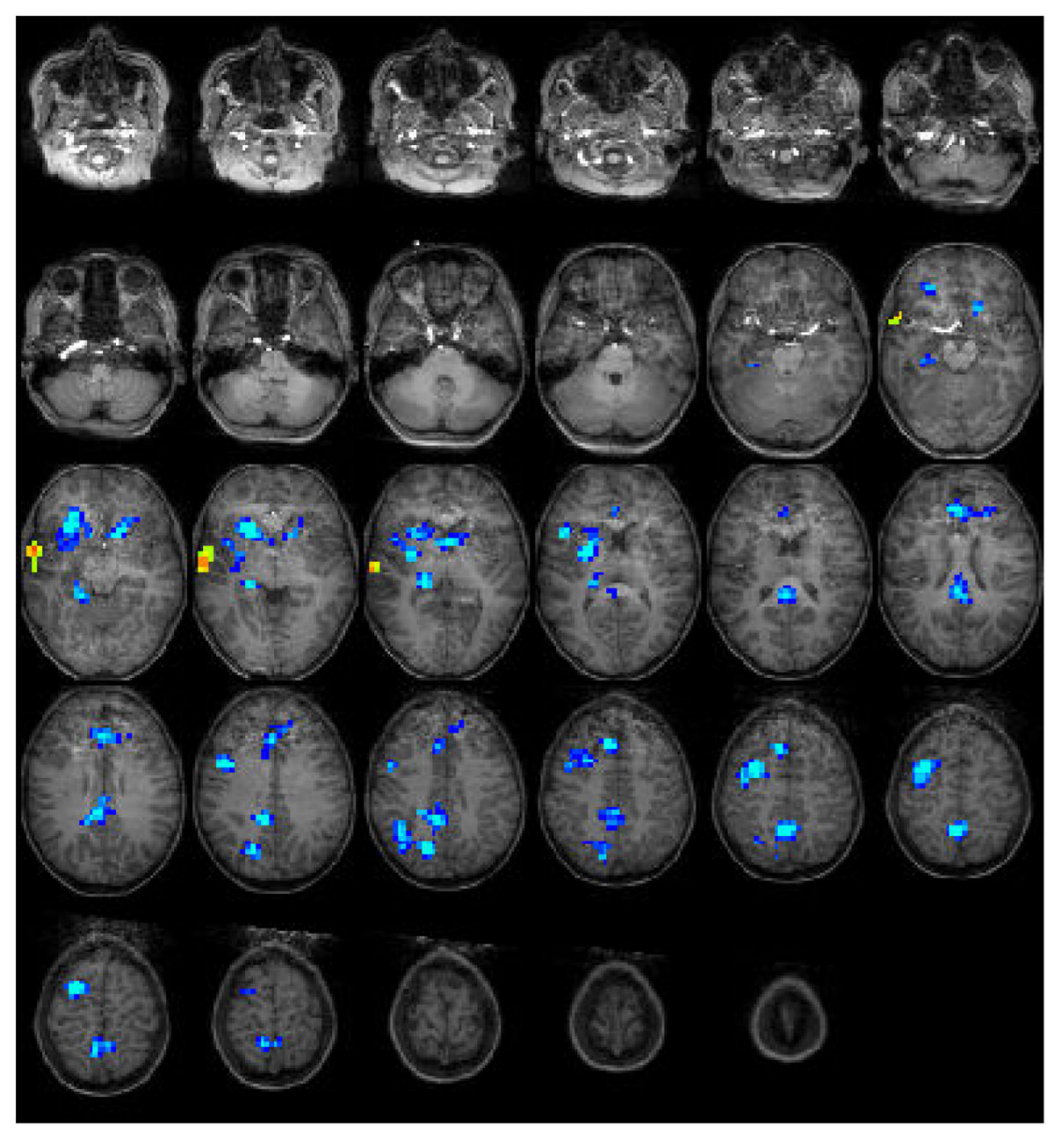
Group composite fMRI activation maps for narrative comprehension in the Test2-middle group [11] year-old children.

For the Test2-middle group (11 years): The statistical parametric map for narrative comprehension>tone listening (Figure 2) was consistent with previous studies using this task [26], with positive activation found in the right middle temporal gyrus (BA 21). Significant deactivation was found in the left medial frontal gyrus (BA 9), right precuneus (BA 7), right cingulate gyrus (BA 23), right inferior parietal lobule (BA 40), right middle frontal gyrus (BA 6), left medial frontal gyrus (BA 8), and four other sub-lobar regions. Talairach coordinates of cluster centroids are listed in Table 1.

**Figure 3:**
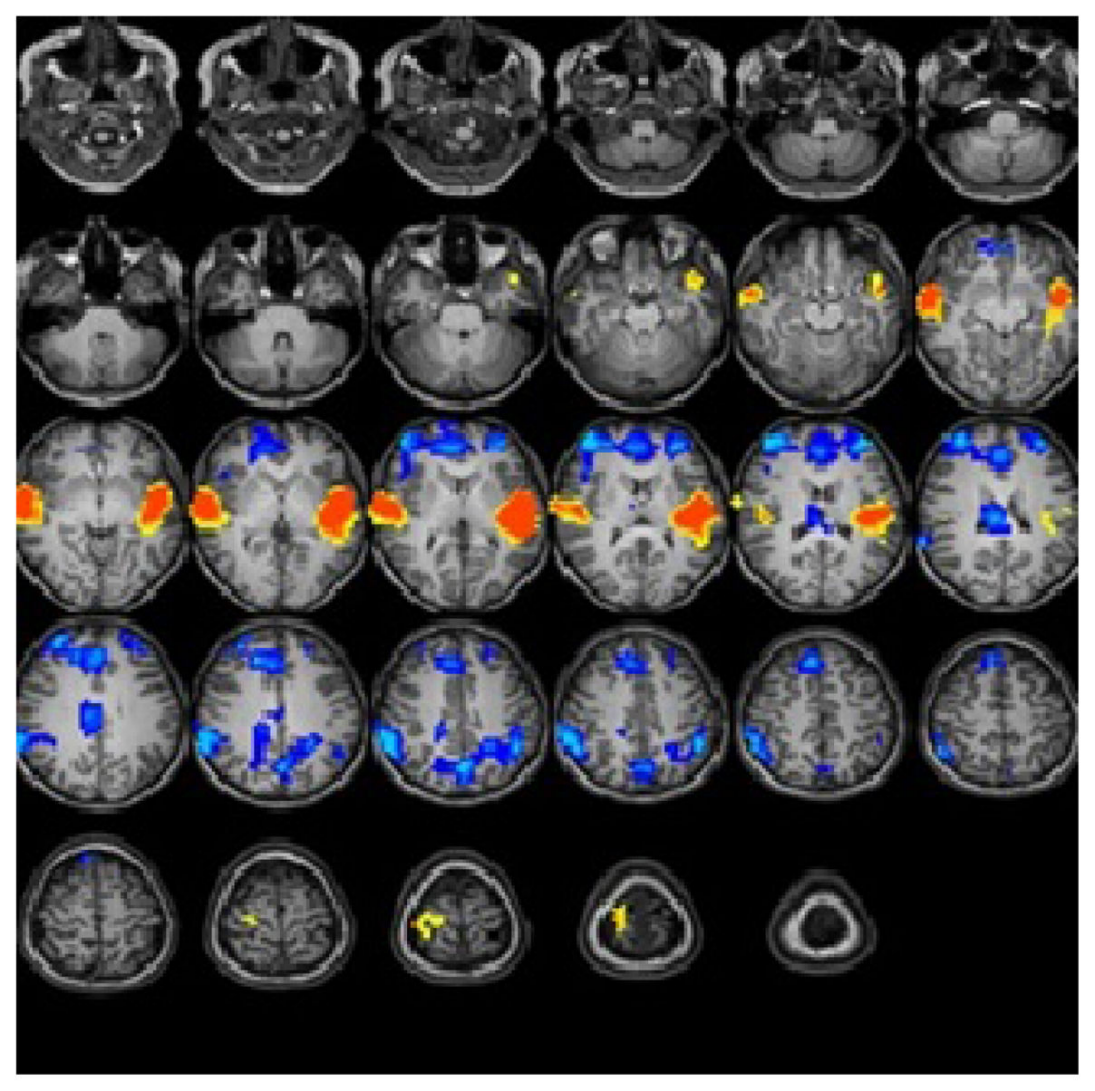
Group composite fMRI activation maps for narrative comprehension in Test3-oldest group [18 year-old children (n=15)].

For the Test-3-oldest group (18 years): The statistical parametric map for narrative comprehension>tone listening (Figure 3) was consistent with previous studies using a similar task [26].

Significant positive activation was found in the right and left STG (BA 22, 38) and in the right middle temporal gyrus (BA 21). Significant deactivation was found in the right and left middle frontal gyrus (BA 10), right anterior cingulate (BA 32), right cingulate gyrus (BA 23), right and left inferior parietal lobule (BA 40), and left precuneus (BA 7). Talairach coordinates of cluster centroids are listed in Table 1.

RM-ANOVA revealed significant differences in deactivation along development in most regions of interest (ROI) defined in the mask (containing the ROI described) in the middle frontal gyrus (BA 10) {F(2,42)=12.1, P<0.001)}, right and left anterior cingulate (BA 32) {F(2,42)=15, P<0.001)}, right middle frontal gyrus (BA 10) {F(2,42)=4.82, P<0.05)}, left inferior parietal lobule (BA 40) {F(2,42)=10.6, P<0.01)}, and right and left precuneus (BA 7) {F(2,42)=19.4, P<0.001)}. No significant differences were revealed in the right inferior parietal lobule (BA 40) {F(2,42)=0.04, P=0.956)}.

Post-hoc analysis suggested that this difference could be attributed to a significant change in deactivation between the Test2-middle (11 years) and Test3-oldest (18 years) groups (P<0.01), as well as a significant difference between the Test1-youngest (5–7 years old) and Test3-oldest groups (P<0.001) in the left middle frontal gyrus (BA 10), left anterior cingulate (BA 32), and right and left precuneus (BA 7). Significant changes were found in deactivation between the Test2-middle and Test3-oldest groups (P<0.05) in the right anterior cingulate (BA 32) and between the Test1-youngest and Test3-oldest groups (P<0.05) in the right middle frontal gyrus (BA 10). Significant increase in deactivation in the left inferior parietal lobule (BA 40) was attributed to a significant increased deactivation between the Test2-middle and Test3-oldest groups (P<0.01) and between the Test1-youngest and Test3-oldest groups (P<0.01).

### Regression analysis

**Figure 4:**
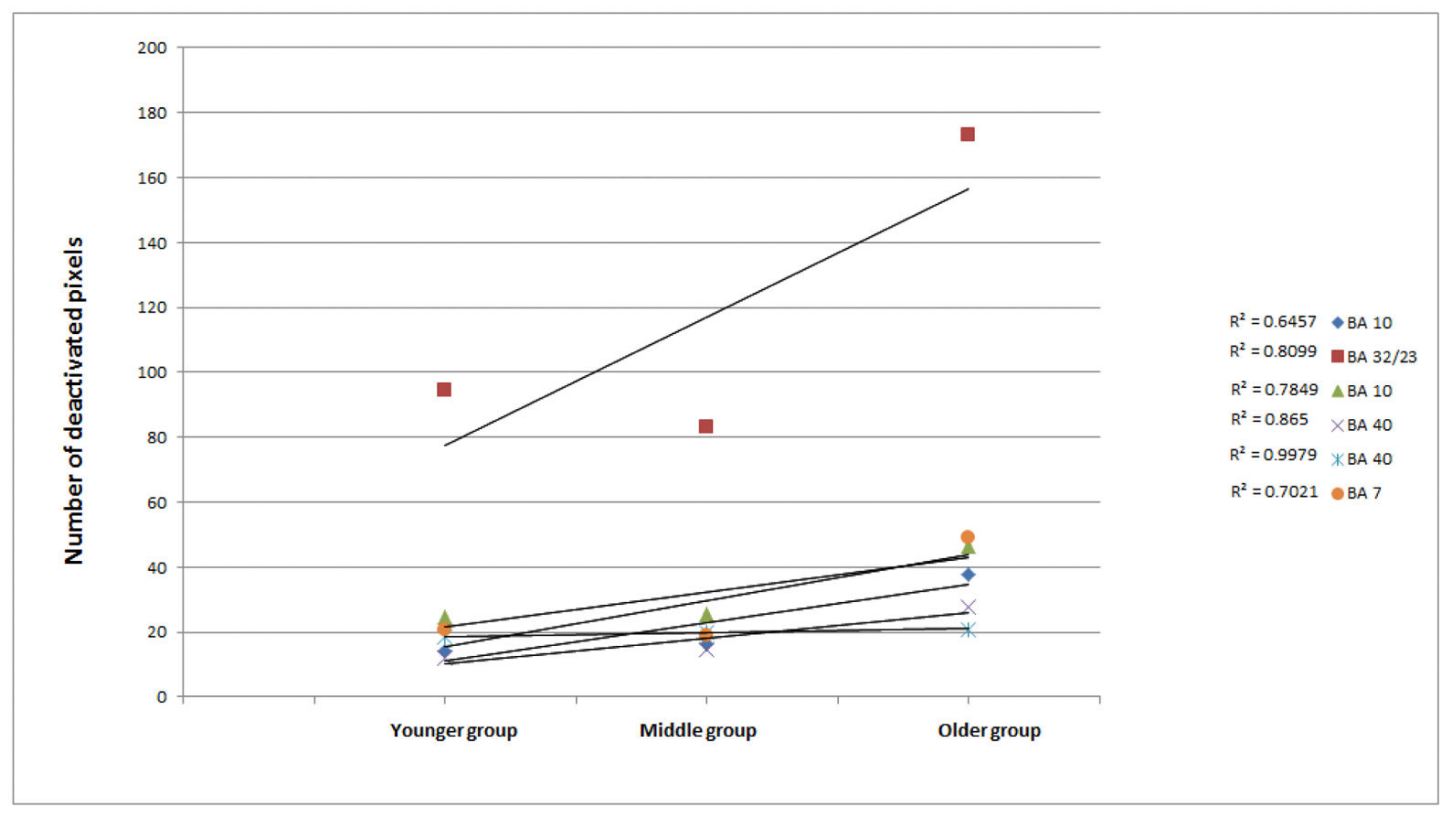
Deactivated areas for the narrative comprehension > tone listening contrast. Number of deactivated voxels for the narrative comprehension > tone listening contrast.

Regression analysis incorporating the three age groups and the number of deactivated voxels in each ROI demonstrated a trend of increased deactivation during aging (Figure 4).

All ROIs that composed the DMN showed a linear increase in the number of deactivated voxels (manifested by a positive slope) along development. Specifically, the ACC (BA32/23) showed the greatest increase in number of deactivated voxels along development. A significant age-related increase in the number of deactivated voxels was found for the frontal ROI [left and right BA 10 (R2=0.81, 0.78, respectively) and BA 32/23 (R2=0.65)] and parietal ROI [left and right BA 40 (R2=0.86, 0.99, respectively) and BA 7 (R2=0.70)]. A Pearson correlation between the narrative comprehension scores and the number of deactivated voxels in the entire sample (N=45) revealed a significant positive correlation between the number of deactivated left voxels in the BA10, ACC (BA32/23), BA40, and left BA 7 and narrative comprehension scores for the Test3-oldest group (r=0.289, P<0.05; r=0.283, P<0.05; r=0.4, P<0.01; r=0.28, P<0.05, respectively). Greater narrative comprehension scores were correlated with more deactivated voxels in the left BAs 10, 40, 32/23, and 7 along development.

## Discussion

The aim of this longitudinal study was to characterize developmental changes of brain regions related to the DMN during a narrative comprehension task. In addition to the well-described increased activation in regions related to auditory and language processing (i.e., the STG) when listening to stories vs to tones [36] results demonstrated increased and more distributed deactivation of the DMN with age and significantly improved behavioral performance from 5 to 18 years of age. Specifically, greater and more distributed deactivation of the DMN in frontal and parietal regions was found in 18 year-olds as compared to the younger ages. Both the Test2-middle and Test3-oldest age groups demonstrated bilateral deactivation, particularly in mid-line components of the DMN in cingulate and pre-frontal regions.

The results of this study support our hypothesis of a developmental change in DMN deactivation while performing a cognitive task accompanied by an increase in behavioral performance. Interestingly, we found that greater deactivation in the left hemisphere and in BA 32/23 was correlated to greater narrative comprehension scores, which might be related to the role of the left hemisphere in language processing [26]. Moreover, Esposito and colleagues also showed increased deactivation in the precuneus and frontal and parietal lobes in 28 (±5) year-old participants while performing the N-back task with different memory loads: the participant recalls stimuli as far back as he or she can remember [44]. The results indicated that greater deactivation of the DMN was related to greater working memory loads, which recruited the anterior more than the posterior cingulate. The increased deactivation of regions within the DMN during a given task may represent a greater attempt of an individual to focus on the content of the task while inhibiting on-going thoughts. In other words, through DMN deactivation, the individual’s brain is able to disconnect from certain distracting internal activity to improve task-focused cognitive function [45]. Therefore, listening to stories at an older age may involve deeper, more associative processing with greater connections to world knowledge, as opposed to simple linguistic processing as at a younger age. This is also supported by neuroimaging studies relating the increased activation in the STG during stories listening [36] and increased intrinsic functional connectivity during rest [46,47] along development to linguistic information processing [36,46]. It was claimed that the region’s activation represents comprehension, causal-temporal ordering of information, and integrative processes during this task [36].

Although the construction-integration model focuses on reading and not on oral-language comprehension, it does provide insights into the differences between a lower-level, bottom-up linguistic processing (construction phase) and a higher-level, top-down processing that involves more-than-basic semantic meaning for presented words/sentences (integration phase) [48]. The construction phase focuses on decoding a single word, during which the semantic meaning of each word is retrieved. The integration phase entails the integration of words into sentences, paragraphs, and stories and is based on previous knowledge and context. Although this model is based on linguistic information from a visual modality (i.e., written language) as opposed to the auditory modality in the current study (i.e., oral language), these two processing phases also may be valid for the current study. Young children might process the verbal information presented in a more “bottom-up” manner with a single meaning for a given word or sentence, whereas older individuals process the narratives easily in this manner, but with greater involvement of world-knowledge and syntactic processing in the “top-down” manner. This may be one reason for the greater deactivation of the ACC in older children, which is involved in conflict monitoring and orienting attention to the task and also was observed by Esposito and colleagues. A failure to deactivate the ACC (and in particular the rostral ACC) has been related to greater error commission and less attendance to the task [12,49]. Additional research comparing different sentence levels and triggering/not triggering world-knowledge associations should examine this point in depth.

Interestingly, the Test2-middle age group appeared to have greater right-sided deactivation in lateral frontal lobe and thalamic components of the DMN than the Test3-oldest group. This finding may suggest the convergence of two different processes: bilateral deactivation of the DMN coupled with task-related activation in the left auditory regions. The contrast of narrative comprehension versus listening to tones may produce some auditory activity specific to the tone stimulus that is not entirely cancelled by activation in the same region during the narrative stream. It may even be that the tone stimulus produces greater activation along development since the participants may be attending the tones or counting them as they grow older (as was previously reported, see [50]. This would contribute to negative blood oxygenation level dependent (BOLD) signal in the contrast of narrative comprehension>tone listening. Such a contribution from the tone stimulus could augment right hemisphere deactivation and give the appearance of right-dominant DMN suppression (Figure 2). In line with that, increased activation to tones at the age of 11 years might be due to increased auditory attention abilities at this age [51], which may result in cancelling out the activation in the left hemisphere. More research using a different contrast specifically and looking the left- activation for tones should be done in order to verify this point.

A possible anatomical explanation for the current study’s results is that changes in regions related to the DMN through development reflect the maturation of the frontal and parietal brain regions and the connections between these regions, as has been suggested previously [52,53]. In their study, Giedd and colleagues observed that the grey-and white-matter maturation in the parietal and frontal regions peaks at 16 years of age, such that the cognitive abilities centered in these regions are mature as well. Changes in cortical grey matter were found to be regionally specific, with developmental curves peaking around age 12 years for the frontal and parietal lobes and around age 16 years for the temporal lobe. However, cortical grey matter continued to increase in the occipital lobe through age 20 years. It has been suggested that cognitive control, which supports inhibition, working memory, planning, and attention, develops throughout adolescence together with the maturation of the frontal and parietal cortices [54–56]. Cognitive control allows the brain to focus on a particular goal by enabling step-by-step thinking while ignoring irrelevant stimuli [56]. These abilities allow a developing child to master basic communication skills, such as narrative comprehension where one must focus attention on a narrative and convert verbal information into memory while incrementally comprehending it. Maturation includes completion of myelination and synaptic pruning. Physiologically, it may also include a process of improving the efficiency of neurovascular coupling between brain activity and the cerebrovascular response needed to increase metabolism to meet the demands of such activity [57]. Based on these known brain-maturation factors, we can infer that to focus on a given task, an older individual will deactivate DMN more efficiently than a younger individual. This may explain the greater deactivation of DMN we observed in the Test3-oldest age group.

In further support of our hypothesis, and together with the DMN deactivation change with age increase, we also found an improvement in narrative comprehension behavioral scores. Since the narrative comprehension score was composed of age-appropriate comprehension questions following the task, these results suggest that to improve comprehension through development, different processes should occur. Based on our results, we postulate that greater deactivation of the DMN is needed in older individuals. A future prospective longitudinal study could verify this point by correlating developmental changes in attention and cognitive measurements with DMN deactivation.

A recent study comparing the developmental changes in BOLD and Arterial-Spin Labeling (ASL) cerebral blood flow (CBF) measured simultaneously in healthy 3–18 year-old children during a narrative comprehension task, suggested an alternative explanation for the increased BOLD signal during development [57]. The rationale for comparing these CBF and BOLD changes to the same stimulus was to determine whether increased BOLD signals as a function of age correspond more closely to either increased metabolic demand associated with neuronal activity or increased CBF [58]. The ASL technique yields a direct estimate of arterial CBF. Using a combined ASL/BOLD acquisition technique, the increase in BOLD signal during the narrative comprehension task with development was not accompanied by a parallel increase in CBF [59]. The authors subsequently demonstrated that the increase in BOLD signal with age is the result of increased neuronal-vascular coupling with development, and not increased neuronal activity. These findings suggest that the increase in DMN deactivation along development, as measured in our study, may be the result of a developmental increase in neural-vascular coupling that causes more efficient deactivation of the DMN. In this case, weaker neuronal- vascular coupling in the Test1-youngest age group of children could mean that suppression of DMN activation is less efficient, resulting in the weaker DMN negative activation we observed in this group. It is therefore still plausible that continued higher levels of activation in the DMN in younger children results in less focus on the narrative comprehension task and poorer performance, as was observed. A future specifically study looking at the deactivation of DMN, rather than at activation, and using a combined ASL/BOLD fMRI acquisition could clarify this point.

## Conclusions

As we hypothesized, our results indicate developmental changes in the DMN, with increasing deactivation of the DMN with age, during a narrative comprehension task. This deactivation pattern along development demonstrates the importance of examining this condition, as well as DMN activation patterns, for a given task. The results may serve as a model for how different pathologies associated with in information processing and task performance act on brain networks. An individual difficulties with attention may process information differently because of an immature deactivation of the DMN, or a child with reading problems might not be able to deactivate this network during the reading process.

## Limitations of the Study

The results should be considered taking into account the following limitations: First, the sample size (n=15) might be restrictive when testing developmental trends. However, the sample was followed longitudinally, which provides additional power in the analysis and support for developmental tendencies, as opposed to cross-sectional testing of the different age groups. Future studies with a larger longitudinal sample should examine this point in depth. Second, the study used the same narratives at the ages of 5–7, 11, and 18 years that were designed for comprehension at the youngest age. Therefore, it may be that greater narrative comprehension scores could be attributed to the fact that the narratives used were very simple for the older children vs the younger ones. However, adopting a task on an age-adjusted level is problematic by means of comparability of the results. A future study should compare the DMN deactivation in both types of tasks, age matched and the same narratives along age, to verify this point. Third, despite the fact that the time difference from the moment the participants heard the stories in the scanner to the moment they were asked the narrative comprehension questions was identical for all participants (so there is no time difference between the groups), there is a possible effect of memory abilities on behavioral change. Since working memory abilities may have an effect on recall abilities, this ability should be evaluated and controlled for across participants. Lastly, since in the current study we were interested in the deactivation in regions related to the DMN, we defined the ROI based on the de-active regions at the age of 18 years. Another way defining of an ROI mask could be to use either a combined map from all time points or a pre-defined DMN mask that might have included other regions active in the Test1-youngest age group that were not revealed by our results.

## Figures and Tables

**Table 1:** Activation/deactivation in the three age groups and centroid coordinates and anatomical location for significant clusters in the narrative comprehension > tone listening contrast.

Group	Negative/positive activation	Region		Cluster			BA
				X	Y	Z	
Test1-youngest	Activation	Left Temporal	Superior temporal gyrus	−51	−15	−1	22
		Right Temporal	Superior temporal gyrus	53	−6	−5	22
Test2-middle	Activation	Right Temporal	Middle temporal gyrus	58	−2	−3	21
	Deactivation	Left Frontal	Medial frontal gyrus	−23	33	21	9
			Medial frontal gyrus	0	28	38	8
		Right Frontal	Middle frontal gyrus	29	5	47	6
		Right Limbic	Cingulate gyrus	3	−34	27	23
		Right Parietal	Precuneus	2	−45	52	7
			Precuneus	36	−51	35	40
		Right Temporal	Middle temporal gyrus	58	−2	−3	21
		Sub-lobar	Caudate	−12	16	1	N/A
			Thalamus	20	−27	0	N/A
			Lentiform nucleus	20	17	2	N/A
Test3-oldest	Activation	Left Temporal	Superior temporal gyrus	−45	−17	3	22
			Superior temporal gyrus	−44	1	−13	38
		Right Temporal	Superior temporal gyrus	56	−11	0	22
			Middle temporal gyrus	56	−3	−11	21
	Deactivation	Left Frontal	Middle frontal gyrus	−27	48	16	10
		Right Frontal	Middle frontal gyrus	35	45	15	10
		Right Limbic	Anterior cingulate cortex	7	39	21	32
			Anterior cingulate gyrus	7	−23	23	23
		Left Parietal	Precuneus	0	−64	35	7
			Inferior parietal lobule	−38	−46	37	40
		Right Parietal	Inferior parietal lobule	53	−43	36	40

BA, Brodmann area
